# Too late or too soon? The replacement gilt paradox

**DOI:** 10.1590/1984-3143-AR2024-0087

**Published:** 2024-10-07

**Authors:** Thomaz Lucia

**Affiliations:** 1 Fibra, Faculdade de Veterinária, Universidade Federal de Pelotas, Pelotas, RS, Brasil

**Keywords:** gilt, puberty, litter size, reproductive failures, removal, lifetime productivity

## Abstract

Due to high annual culling rates, pig farms require a constant income of replacement gilts. Gilts typically reach puberty at nearly six months of age. Puberty may be induced through early boar exposure, therapy with steroid hormones and chorionic gonadotropins, and optimized by identifying biological predictors and risk factors. Old age at the time of the first mating is associated with an increased risk of premature culling, often attributed to reproductive failures and locomotor problems. While female prolifacy has increased substantially during the last few decades, selecting for litter size to optimize lifetime productivity would be more efficient after two parities. Additionally, uterine capacity and the number of functional teats should be considered in selecting future dams. For each female, the cost-effective number of parities at removal is determined by the cumulative number of pigs born and weaned during the total herd days.

## Introduction

The swine industry has continuously challenged farms to enhance reproductive efficiency, since increased performance yields more kilograms of pork and higher revenues. Recent benchmarks (Agriness®, 2023; PigCHAMP®, 2023) have revealed that highly productive herds reached unprecedent (so far…) reproductive efficiency, with more than 30 pigs weaned per female per year (PW/F/Y). Considering the biological potential of swine females (Koketsu et al., 2017), the reproductive efficiency is expected to continue increasing in the coming years. The increase in reproductive efficiency has been driven by a remarkable increase in female prolifacy, due to genetic improvement on ovulation rate. Over the past decades, ovulation rates have increased by nearly 0.2 oocytes per year for both gilts and sows (Kemp et al., 2018). Hence, gilts have made substantial contribution to enhance reproductive efficiency, with the total litter size on the first farrowing increasing by at least three additional piglets.

Replacement gilts are the raw material upon which the foundation of the breeding herd is built. Ideally, gilts would be bred as young as possible, consistently farrow and wean large litters, and be quickly rebred in several consecutive reproductive cycles during a long lifetime. Gilts from most genetic lineages reach puberty within 150-180 d of age, which can be stimulated through boar exposure starting with 140-160 d of age (Breen et al., 2005; Knox et al., 2021). Although puberty is signaled by the first estrus expression, regular cyclicity is established when the first estrus is followed by an ovulation and a subsequent estrus within a physiological interval of approximately 21 d. Regular estrus cycles are characterized by a follicular phase with endocrine predominance of estrogen during proestrus and estrus and by a luteal phase with endocrine predominance of progesterone during metestrus an diestrus (Soede et al., 2011).

After puberty, gilts may be bred for the first time at either their second or third estrus. Ideally, gilts should have a backfat thickness of 13-15 mm when first mated (Bortolozzo et al., 2023). However, they may be bred regardless of backfat thickness when their liveweight is between 135 and 150 kg (Patterson and Foxcroft, 2019; Faccin et al., 2022). Depending on their growth rate, gilts either too young (less than 180 d old) or too old (more than 220 d old) at the first mating may be overweight, which increases the probability of reproductive failures that may shorten their reproductive lifetime (Tummaruk et al., 2007; Schenkel et al., 2010).

With annual culling rates near to 40-45% (Agriness®, 2023; PigCHAMP®, 2023), the breeding herd needs a constant supply of high-quality replacement gilts to ensure inventory stability and to maintain performance targets (Patterson and Foxcroft, 2019). However, the culling of gilts that never farrowed may be substantial (Lucia et al., 2000a; Engblom et al., 2007) and all culled females are replaced by other gilts. Thus, the replacement gilt pool commonly represents a considerable proportion of the breeding herd, and, for some farms, maintaining a stable parity distribution may be challenging.

Since gilts that exhibit the first estrus at early ages accumulate fewer nonproductive days (NPD) prior to the first service and are more likely to have greater lifetime productivity than those reaching the first estrus at older ages (Patterson et al., 2010; Patterson and Foxcroft, 2019), efficient methods to induce puberty are of interest. Thus, additional questions arise: Is it feasible for teenage gilts to reach puberty, gestate, farrow, lactate, remain productive throughout consecutive parities and retire with prolonged longevity? Can gilts start the reproductive life at decreasingly younger ages and have increasingly longer lifetimes? The present article addresses some aspects of this paradox.

## Puberty: the earlier the better?

While they are still baby pigs inside the uterus of their mothers, gilts already possess enough oocytes in their ovaries to sustain all subsequent reproductive cycles that may occur throughout their lifetime. These oocytes reside in primordial follicles, which are predominant in the ovaries until nearly 90 d after birth (Christenson et al., 1985). Although most primordial follicles remain quiescent, some are activated by nearly 70 d of prenatal development and differentiate into primary follicles; these will either progress into secondary (pre-antral) follicles or undergo atresia. Secondary follicles can be observed prenatally in the ovarian surface as birth approaches (Knox, 2023). During the prenatal period, the number of hypothalamic neurons capable of secreting GnRH is already established, but their functionality is not yet fully developed due to their high sensitivity to an estradiol-mediated negative feedback mechanism.

When prepubertal gilts are 60-90 d old, their ovaries become responsive to gonadotropins and tertiary antral follicles are formed (Knox, 2023). However, prior to puberty, antral follicles are in limited number, do not progress to the ovulatory stage and undergo atresia. This occurs due to an unfavorable endocrine environment, since the neurons on the tonic center of the hypothalamus exhibit limited capacity to respond to stimuli through connections with presynaptic neurons, while the neurons on the surge center of the hypothalamus remain unresponsive. As the GnRH release occurs in pulses with low frequency and high amplitude during the prepubertal period, its circulatory concentration is not sufficiently high to stimulate gonadotropin release.

Puberty approaches as the sensitivity of the hypothalamic neurons to the estradiol-mediated negative feedback gradually declines. Consequently, stimuli from environmental cues and from metabolic signalers with increasingly circulatory concentrations are processed more efficiently through interactions among GnRH-secreting neurons and presynaptic neurons (Lents et al., 2020). As the responsiveness of the hypothalamic neurons is enhanced, GnRH pulses become more frequent and narrower, resulting in increased LH release from the hypophysis, thereby stimulating estradiol secretion by the ovarian follicles. The heightened estradiol circulatory concentration stimulates hypothalamic neurons from the surge center, which were previously unresponsive during the prepubertal period, promoting an increase in GnRH release. This, in turn, amplifies the release of gonadotropins by the hypophysis and estradiol by the ovaries, characterizing a positive feedback mechanism (Knox, 2023).

Under such endocrine environment, a pool of small antral follicles present in the ovaries is recruited (Soede et al., 2011), influenced by the FSH processed by receptors located on granulosa cells. Some of these follicles will grow and eventually ovulate, while others will become atretic. Thereafter, receptors located in the theca are also activated, enabling growing follicles to become responsive to LH. The theca cells increase their endocrine activity to convert cholesterol into androgen steroids, which will be later converted into estradiol in the granulosa. The increased estradiol concentration induces characteristic behaviors expressed by gilts during the follicular phase of the estrous cycle: agitation, mounting other gilts when housed in collective barns, and swelling of the vulva, during proestrus; and lordosis signaling acceptance of a mount (which can be identified by back pressure, either in contact with a boar or induced by a trained technician), during estrus (Langendijk et al., 2000; Tummaruk et al., 2007). At ovarian level, the follicles increase in diameter and the fluid accumulated in the antrum pressures the follicular wall until its rupture, leading to the release of the oocytes during the first ovulation. Although ovulation generally occurs at the final third part of the estrus period (Nissen et al., 1997; Soede et al., 2011), compared to older sows, gilts may have shorter estrus and less characteristic estrus signs.

After ovulation, several corpora lutea are formed at the ovarian surface, secreting progesterone, presumably to support a pregnancy. However, the first ovulation generally does not result in pregnancy since pubertal gilts would still need to wait until either their second or third estrus to be bred for the first time, depending on their liveweight (Patterson and Foxcroft, 2019; Faccin et al., 2022; Bortolozzo et al., 2023). Therefore, the function of the corpora lutea declines approximately two weeks after the ovulation, due to the action of the prostaglandin F2α (PGF2α) released from the endometrium, resulting in luteolysis (Soede et al., 2011; Rensis et al., 2012). Then, the circulatory progesterone concentration decreases, creating an endocrine environment suitable for the establishment of a subsequent estrus cycle.

## Family values

The selection for increased ovulation rates may have indirectly selected females prone to farrow lighter piglets, as larger litters are commonly heterogenous, including piglets with poor intrauterine growth and low birthweight (Alvarenga et al., 2013; Declerck et al., 2016). This occurs more often in litters from primiparous than from multiparous sows (Craig et al., 2017) and may be repeatable across parities (Patterson et al., 2020). Therefore, gilts born to primiparous mothers may be lighter and have an inferior growth rate compared to those born to multiparous mothers (Wijesiriwardana et al., 2022) and may have high backfat at the time of selection for replacement (at nearly 24 weeks of age), indicating lower muscle body deposition compared to fat deposition (Magnabosco et al., 2016, Almeida et al., 2017a). Low weight at both birth and puberty and high backfat at puberty are undesirable, incurring in increased risk of reproductive failure and premature culling and reduced litter size at subsequent pregnancies (Tummaruk et al., 2007; Kummer et al., 2009; Magnabosco et al., 2016). However, although low birthweight gilts may exhibit impaired ovarian follicular development (Almeida et al., 2017a), low birthweight seems unrelated to age of puberty (Almeida et al., 2017b; Patterson et al., 2020). When low birthweight gilts, from either primiparous or large litters, are fostered into other litters, their postnatal growth rate and weaning weight may be similar comparable to gilts kept in their original litters (Almeida et al., 2017b; Magnabosco et al., 2016). Thus, puberty tends to occur earlier for gilts with fast growth (Kummer et al., 2009; Patterson et al., 2020). Nevertheless, this assumption may be confounded by the fact that gilts from small litters (also more common for primiparous dams) commonly have high birth weight and may grow more slowly during lactation, due to less competition for suckling (Vallet et al., 2016). Additionally, the weak correlation between birth weight and age at puberty suggests that gilts selected for growth rate may not necessarily achieve puberty at earlier ages (See et al., 2018). Taken together, those findings suggest that gilts from litters farrowed by primiparous dams may not be the optimal choices for future herd replacement compared to gilts from litters farrowed by multiparous sows (Craig et al., 2017; Wijesiriwardana et al., 2022). For gilts from primiparous dams, the preweaning growth rate rather than the birth weight may be a more accurate predictor of age at puberty, thereby having a positive effect on subsequent gilt retention rates (Knauer et al., 2010).

Low birth weight and preweaning growth may occur in progenies from primiparous dams because gilts selected for growth rate and early puberty only complete their maturation between the first and the second parities (Schenkel et al., 2010; Ocepek et al., 2016). Thus, if daughters of a given dam are to be selected as replacement gilts, that would be more appropriate within the second and third parities. On the other hand, this may contrast with the findings that, although primiparous may farrow and wean lighter piglets, their progeny may still present subsequent compensatory growth, reaching similar weight and age at puberty compared to progenies from multiparous (Almeida et al., 2017b; Magnabosco et al., 2016). However, a genome-wide association study (GWAS) reported moderate genetic correlations between growth rate and age at puberty characterized by a quadratic association, suggesting that, within contemporary cohorts, gilts with both the slowest and the fastest growth rates would be less appropriate for breeding herd replacement (Wijesena et al., 2023). Thus, efficient selection for age at puberty and growth rate should be conducted separately for each trait.

## Inheritance and succession

Data on certain events that occur way before puberty or during the gilts’ infancy are currently being screened to identify predictors of their potential future performance. Twelve single-nucleotide polymorphisms associated with delayed puberty in gilts that failed to display estrus after 240 d of age were identified in a GWAS, suggesting that genetic markers are promising tools for early identification of gilts unfit for breeding herd replacement (Nonneman et al., 2014).

Some physical traits of the reproductive organs of gilts are potentially associated with their future reproductive performance. Vaginal length, measured at the time of slaughter, was found to be longer for gilts with higher birth and body weights than for lighter gilts, although associations with the development of the entire genital tract were not evident (Almeida et al., 2017a). A vulva width score, measured at nearly 15 weeks of age, identified morphometric variations between proestrus and estrus, especially 24 hours prior to estrus onset (de la Cruz-Vigo et al., 2022). Moreover, high vulva scores may be associated with early estrus expression (Graves et al., 2019) and total litter size (Romoser et al., 2019). However, as such associations were marginal, the vulva score is not considered a reliable predictor of reproductive performance (Mills et al., 2020), especially because there is currently no information about its potential heritability. Nonetheless, gilts can respond positively to selection for reproductive tract development. Gilts selected for uterine capacity across several generations showed improvement in the subsequent born alive litter size and prenatal piglet survival, despite a slight reduction in ovulation rate, and their piglets presented similar birth and weaning weights compared to those farrowed by unselected control gilts (Freking et al., 2007). Such effects were also associated with improved retention rates at subsequent parities (Freking et al., 2016). Thus, gilts selected for uterine capacity would likely farrow and wean large litters during a prolonged lifetime.

Although the selection of replacement gilts with at least 14 functional teats has been employed for many years (Kim et al., 2005) and the number of available teats increased over time (Drake et al., 2008), such number still falls short of the current average litter size. This limitation may restrict the availability of colostrum and milk to piglets when nursing large litters (Faccin et al., 2022), thus increasing the risk of occurrence of runts and preweaning mortality (Devillers et al., 2011; Declerck et al., 2016). Therefore, suckling gilts with inadequate colostrum intake would not be suitable choices for future replacement, as they would exhibit impaired subsequent performance.

Some biomarkers may help forecasting the future reproductive performance of gilts, even when detected at very young ages. Distinct lipidomic profiles in samples of the anterior vagina content collected by swabs at 21 d of age were identified in gilts with similar birth weight, vulva width, and average daily weight gain (Mills et al., 2021). Thereafter, these gilts were categorized as either highly fertile or infertile, based on their estrus expression and the number of piglets weaned across two parities. A distinct relative abundance of some fatty acids was identified across these categories. Some of these fatty acids may be present in steroid hormones, acting as pherohormones released by females in secretions such as saliva, urine, and feces, to communicate their endocrine status to boars (Devillers et al., 2004; Sankarganesh et al., 2021). Once identified by boars through olfactory stimulus, these pherohormones bind to carrier proteins in the systemic circulation and are related to characteristic behaviors, such as hypersalivation and sniffing (Silambarasan et al., 2019; Sankarganesh et al., 2021), which help to stimulate puberty onset. However, the stimuli of pherohormones in urine is less effective in gilts and non-estrous females compared to sows in estrous (Silambarasan et al., 2019). Potential biomarkers of boar receptivity were also detected through metabolomic analyses of salivary samples of prepubertal gilts (Goudet et al., 2019, 2021), which may help determining the optimal boar exposure period.

In gilts treated with chorionic gonadotropins (eCG and hCG) between 60 and 100 d of age, increased serum estrogen concentration was correlated with increased frequency of stillbirths and reduced born alive litter size throughout the first three parities, whereas increased serum concentration of anti-Mullerian hormone (AMH) was correlated with prolonged gestation length (Steel et al., 2018). As basal estrogen concentration contributes to accelerate puberty and regular cyclicity by weakening the negative feedback mechanism on the hypothalamus-hypophysis-gonads axis, assessing serum estrogen concentration at very young ages might aid in selecting replacement gilts. However, despite of its association with the establishment of the population of antral follicles at the ovaries, as reported in ruminants (Batista et al., 2014; Torres-Rovira et al., 2014), the role of AMH as a predictor of puberty in gilts is still unclear.

## Drugs in youth

Although pig farms worked with weekly weaning intervals for decades, many switched to batch farrowing systems (Lurette et al., 2008; Corezzolla et al., 2020), with groups of served females at longer intervals (e.g., 14, 21 or 28 d). Thus, the use of drugs may be necessary to induce synchronized puberty in large groups of replacement gilts.

Exogenous progestogen supplementation is commonly used to control the estrous cycle in ruminants (Bó et al., 2016; Miranda et al., 2018). These treatments involve the slow release of progestogens through intravaginal devices to mimic the action of natural corpora lutea. This promotes negative feedback on GnRH by downregulating estrogen receptors in the hypothalamus, thereby suppressing LH release and estrus expression. Afterwards, upon removal of such devices, treatment with analogues of PGF2α and estradiol esters is used to induce luteolysis and ovulation. However, in swine, the use of intravaginal devices has yielded unsatisfactory results (Ulguim et al., 2019; Quirino et al., 2020), and currently, there are no commercially available devices for use in pigs.

Daily oral administration of a progestogen for 14-18 d can be used to induce and synchronize estrus in gilts (Estienne and Crawford, 2015; Ravagnani et al., 2020). In prepubertal gilts, this treatment enhances the number of antral follicles, the responsiveness of the granulosa cells to FSH and ovarian steroidogenesis (Wang et al., 2018; Ziecik et al., 2020), resulting in estrus expression in most treated gilts within 2-3 d after the withdrawal. Although the ovulation rate may be increased, benefits on litter size may not occur since the number of embryos may be unaltered (Ravagnani et al., 2020). The synchronized cyclicity resulting from the exogenous progestogen supplementation is important for transitioning herds from conventional weekly flow to batch farrowing management (Corezzolla et al., 2020), but inconsistent results may occur due to the labor-intensive nature of the daily supplementation and individual variations in progestogen consumption (Kaeoket, 2008; Werlang et al., 2011). Moreover, such therapy may be associated with the occurrence of ovarian cysts, especially in prepubertal gilts (Ziecik et al., 2020).

Considering these limitations, injectable drugs may be suitable alternatives. Parenteral supplementation with long-acting progesterone, successfully used in beef heifers (Lima et al., 2020) and anestrous ewes (Ungerfeld and Freitas-de-Melo, 2023), has been tested in pigs. In pregnant swine females, this treatment has shown positive effects on endometrial secretions and embryo survival (Muro et al., 2020; Szymanska and Blitek, 2020), since progesterone naturally stimulates the vascular development and proliferation of both the luminal and glandular uterine epithelium (Bailey et al., 2010). Nonetheless, data about the efficiency of such treatment on puberty induction are still limited. Treatment with long-acting progesterone was ineffective to induce cyclicity in prepubertal gilts (Baldessar et al., 2023), likely because the metabolic condition of those gilts was insufficient to remove the negative feedback mechanism that inhibited GnRH release (Lents et al., 2020). This suggests that this treatment may be more effective in older and heavier gilts. In peripubertal gilts approaching natural estrus, the exogenous supplementation compounded the endogenous progesterone synthesis, suppressing LH release and follicle development, and delaying estrus expression (Baldessar et al., 2023). A prolonged luteal phase of the estrous cycle may be useful to synchronize subsequent estrus expression of gilts in farms working with batch farrowing systems.

The luteal phase of the estrous cycle can also be prolonged through exogenous supplementation with estrogens. In cycling females, estrogen treatment 10-13 d after estrus expression would mimic the mechanism of maternal pregnancy recognition (Zavy et al., 1988; Noguchi et al., 2010), which is physiologically signaled through endogenous estrogen production by the developing embryos inside the uterus (Waclawik et al., 2017). This pseudo-pregnancy endocrine environment would maintain high circulatory progesterone concentration until the administration of a PGF2α analogue 10-15 d later, to promote luteolysis (Guthrie and Polge, 1978; Rensis et al., 2012). Thus, the beginning of a new estrous cycle would be synchronized, with no detrimental effects on subsequent follicular development and ovulation (Noguchi et al., 2011), embryo recovery rate (Leal et al., 2022), and farrowing rate and litter size (Noguchi et al., 2010). Recently, estrogen treatment to induce pseudopregnancy was adapted to induce lactation in non-pregnant gilts (Cordeiro et al., 2023), suggesting that gilts not destined to replacement might be used as milk suppliers in farrowing rooms, since modern sows frequently farrow litter sizes that exceed their number of teats. However, such approach may be limited due to concerns from relevant segments of the public opinion about potential side effects of steroid hormones residues (Qin et al., 2004; Ganmaa and Sato, 2005).

Treatment with the eCG/hCG combination may be a non-steroidal alternative to synchronize estrous in cyclic gilts. As the responsiveness of the corpora lutea only occurs after 12 d post-ovulation, treating gilts in diestrus with chorionic gonadotropins within a 72-h period would extend the luteal phase of the estrous cycle (Guthrie and Polge, 1978). The increased diameter of the ovarian follicles induced by eCG, coupled with the luteotrophic activity of hCG would maintain a high circulatory progesterone concentration (Soede et al., 2001; Ziecik et al., 2018). Thereafter, a luteolytic drug would be administered to induce synchronized estrus expression (Guthrie and Polge, 1978; Rensis et al., 2012). With this management, the interval between consecutive estrous may be extended until nearly 30 d (Soede et al., 2001), which aligns with farms employing batch farrowing management. Even though treatment with eCG/hCG is widely used in females with delayed estrus expression, especially in weaned primiparous (Vargas et al., 2006; Kraeling and Webel, 2015), its use in gilts with delayed estrus has led to disappointing results (Breen et al., 2005; Stančić et al., 2012). That may have occurred due to the occurrence of ovarian cysts (Kirkwood, 1999) and because a substantial proportion of the treated gilts may have had previously undetected estrus (Stančić et al., 2012), resulting in unnecessary costs with hormones. Treatment with eCG/hCG may synchronize estrus expression in both prepubertal and pubertal gilts with unknown cyclicity which were previously supplemented with exogenous progestogens, although that approach is not frequently employed (Estienne and Crawford, 2015).

A prolonged luteal phase can also be induced in cyclic gilts by a double hCG treatment 12-15 d after estrus expression (Brito et al., 2024). This treatment would promote luteinization of growing follicles and the formation of accessory corpora lutea, resulting in circulatory progesterone concentration comparable to that of natural cycles (Guthrie and Polge, 1978; Soede et al., 2001). This hCG treatment resulted in prolonged inter-estrus interval and concentrated estrus expression within at most 4 d, but it was followed by a reduction in the subsequent litter size (Brito et al., 2024). At this point, this would prevent the routine use of such treatment in farms using batch farrowing systems, pending adjustments on the hCG doses and the treatment period.

Alternatively, contraceptive drugs can be used to induce the production of anti-GnRH antibodies, impairing LH release by the hypophysis and steroid synthesis by the gonads (Oliviero et al., 2016; Squires et al., 2020). Such drugs were originally developed for use in boars, benefitting their welfare since surgical castration becomes unnecessary (Bonneau and Weiler, 2019; Faggion et al., 2023), and improving feed efficiency and carcass quality due to the increased deposition of lean muscle tissue and reduction in boar taint (Oliviero et al., 2016; Squires et al., 2020). In gilts, along with improvements in feed efficiency and carcass quality (Daza et al., 2014; Poulsen Nautrup et al., 2020), the use of contraceptive drugs resulted in reduced estrous occurrence (Bohrer et al., 2014). Since a relevant number of candidate genes related to the expression of sexual steroids and metabolites associated with boar taint have been identified (Squires et al., 2020; Faggion et al., 2023), anti-GnRH drugs may eventually be used to modulate female puberty and cyclicity, as observed in heifer calves (Hernandez-Medrano et al., 2013; Schütz et al., 2021), and to heal ovarian malfunctions, as observed in cows with chronic ovarian cystic disease, who resumed cyclicity after treatment (Viana et al., 2021).

## Rookie mothers: collateral effects of teenage pregnancies

Gilts that reach puberty at young ages will become mothers at young ages as well. Puberty attainment requires multifactorial stimuli until a physiological balance between physical and reproductive development is reached (Beltranena et al., 1991; Evans and O’Doherty, 2001). During the prepubertal period, metabolic input prioritizes physical development. Thereafter, the basal metabolic demand shifts towards maintaining physical condition, while an energy surplus is stored as adipose tissue, which may serve as a source of various metabolites, including fatty acids and glucose. In gilts with adequate fat deposition, that is signaled to pre-synaptic hypothalamic neurons through an increase in leptin secretion by the adipose tissue (Blüher and Mantzoros, 2007), boosting GnRH synthesis and release (Barb et al., 2008; Lents et al., 2020). In cyclic females, increased presence of leptin and its receptor was detected in growing follicles and recently formed corpora lutea (Gregoraszczuk et al., 2007; Moreira et al., 2014). However, consumer demand for low-fat animal protein has directed selection programs of modern pig genetics towards reduced voluntary feed intake and conversion, resulting in animals with robust lean tissue mass and reduced fat deposition. Thus, fast-growing gilts reaching puberty at very young ages may have reduced adipose tissue reserve (Beltranena et al., 1991; Kummer et al., 2009). That might be alleviated by supplementing gilts with polyunsaturated fatty acids early in life (after weaning), which has been found to be related to improved feed efficiency and reduced circulatory cholesterol concentrations (Otte et al., 2019). However, potential effects on puberty onset are uncertain because benefits on gene expression for the LH receptor and steroidogenic enzymes were not observed. Furthermore, young primiparous may have diminished capacity to nurture their first litter and to overcome the metabolic challenge of the first lactation, since they need to continue growing until maturity (Schenkel et al., 2010; Ocepek et al., 2016).

Motherhood is tough. Since gilts are unfamiliar with farrowing rooms, their first contact with farrowing crates may trigger physiological stress, as indicated by the raised plasma cortisol concentration observed for females in crates (Jarvis et al., 2001). This stress can result in behavioral changes harmful for the health and welfare of both the dam and the litter (Ahlström et al., 2002; van Rens and van der Lende, 2004). Although data relating pregnancies at very young ages to subsequent reproductive performance are still not widely available for pigs, data from murine experimental models have shown a reduction in litter size in subsequent parities (Yang et al., 2021), attributed to abnormal embryo implantation and placental development during initial gestational periods and altered gene expression at uterine level.

The perinatal period is naturally stressful for all females (Kranendonk et al., 2008; Rutherford et al., 2012) and farrowing itself is a painful event due to the endogenous release of oxytocin, which stimulates intense uterine contractions to facilitate the expulsion of piglets (Nagel et al., 2019). These situations are particularly challenging for first-time mothers (Mainau and Manteca, 2011; Ward et al., 2022). The stress and the pain may cause females to be restless, especially during the initial stages of expulsion, which can endanger the piglets (Chen et al., 2008; Mainau and Manteca, 2011). Although primiparous may eventually express aggressiveness and try to savage the piglets during farrowing (Ahlström et al., 2002; van Rens and van der Lende, 2004; Chen et al., 2008), that behavior appears to be unrelated to poor maternal ability and tends to cease with the increase in the contact with the newborns and during suckling. Piglet postnatal survival can be improved with farrowing supervision (Holyoake et al., 1995; Vallet and Miles, 2017), which is limited in intensively managed farms by the reduced labor availability. Under field conditions, it is common to administer a PGF2α analogue to the periparturient females nearly 72 h prior to the scheduled day of farrowing to prevent farrowing outside working hours. This treatment accelerates labor by impairing the function of corpora lutea and reducing the circulatory progesterone concentration (Rensis et al., 2012; Vallet and Miles, 2017). Nevertheless, the effects of anticipating the stress of farrowing on primiparous females is still to be determined. Additionally, besides its obstetric use in females lacking uterine contractions, exogenous oxytocin treatment may be used to shorten the duration of ongoing farrowings, which may result in an increased stillbirth rate (Lucia et al., 2002; Borges et al., 2005).

With the increase in the first litter size, the duration of farrowing also increased for primiparous females, which may prolong their distress (Vanderhaeghe et al., 2013; Ward et al., 2020). Females may be treated with glucocorticoids and non-steroidal anti-inflammatory drugs prior to farrowing, which can increase the uterine muscular tone and improve the transition of an atonic myometrium to a contractile stage, mitigating pain and discomfort (Kashanian et al., 2008; Bhaumik et al., 2023). Although this treatment can reduce farrowing duration and the need of obstetric intervention, no benefits on stillbirth rates and piglet survival were observed (Will et al., 2023). For primiparous, the risk of stillbirths may be exacerbated by the fact that their birth canal is narrower compared to multiparous sows (Hoving et al., 2010; Ocepek et al., 2016).

Primiparous females can also experience stress after farrowing due to their unfamiliarity with being suckled by piglets, which may have negative effects on postnatal survival, especially for piglets with low viability and reduced colostrum and milk intake (Devillers et al., 2011; Declerck et al., 2016). Several management strategies can optimize piglet survival in farrowing rooms: cross-fostering (Alexopoulos et al., 2018); split weaning (Zak et al., 2008); intermittent suckling (Gerritsen et al., 2009); and the use of nurse sows (Baxter et al., 2013). Nonetheless, the efficiency of all these practices heavily relies on human labor, which is increasingly limited in modern farms.

Life can be cruel for primiparous females. After their first farrowing and lactation, they have little time to mourn the separation from their litters at weaning. Their immediate task is to return to estrus as soon as possible to initiate a new reproductive cycle. Typically, swine females remain in anestrus during lactation, thanks to the frequent stimuli on the mammary glands by the piglets, which maintains a high circulating oxytocin concentration, promoting a negative feedback mechanism on GnRH and LH release (Soede et al., 2011). After weaning, the pattern of GnRH release is restored, allowing the follicles that were growing to mature until ovulation (Kemp et al., 2018). Presently, the weaning-to-estrus interval (WEI) is within 3-6 d for 80-90% of the weaned females, suggesting that the follicles destined to ovulate were recruited almost simultaneously with the weaning (van den Brand et al., 2000; Kemp et al., 2018). However, for females with a long WEI, the LH pulses are irregular and insufficient to stimulate the growth and maturation of the available follicles, which may lead to short estrus duration and less characteristic estrus signs (Kemp and Soede, 1996; Lucia et al., 1999a). Unfortunately, a significant proportion of these females with long WEI may be primiparous, as their risk of delayed estrus expression post-weaning is greater compared to mid-parity sows (Koketsu and Dial, 1997; Koketsu et al., 2017). This delay may result from high lactational catabolism (Koketsu and Dial, 1997; Prunier et al., 2010) since primiparous females have not yet completed their physical development.

Consequently, sophomore females may be prone to facing the second-litter syndrome, particularly if they were either too young or too light at puberty (Hoving et al., 2010; Sell-Kubiak et al., 2021), which increases the risk of premature culling (Engblom et al., 2007). The impact of the second-litter syndrome can be mitigated by optimizing sow weight gain from the first breeding to weaning and by avoiding tight selection pressure on first-parity litter size (Thingnes et al., 2015; Wülbers-Mindermann et al., 2015). This approach may involve increasing the retention of primiparous females with suboptimal litter size and transferring the selection pressure for litter size to the following parity. Nonetheless, primiparous females that farrow a large first litter and do not have impaired performance in the second parity generally continue to farrow above-average subsequent litter sizes (Sasaki et al., 2011; Iida and Koketsu 2015). Therefore, the born alive litter size in the first two parities could be an indicator of subsequent reproductive performance due to its high heritability and positive association with retention rates and lifetime productivity (Gruhot et al., 2017; Freyer 2018).

## Retirement: is it too early?

Historically, reproductive failures are the leading reasons for culling females of all parities, and particularly relevant among gilts and primiparous sows (Lucia et al., 2000b; Engblom et al., 2007). That did not change over the years, despite improvements in ovulation rate, litter size and WEI. Among the risk factors contributing to culling due to reproductive failures, late age of first mating is considered the most relevant (Koketsu et al., 2020). Gilts experiencing delayed first mating often return to estrus, after either long or irregular intervals (Tani et al., 2016). Even when successfully bred and carrying the gestation to term, gilts mated at an older age may have prolonged WEI (Saito et al., 2011). Thus, gilts with a late first mating are more likely to be retired soon.

Common health conditions such as limb and claw lesions can progress to serious locomotor problems and lameness, significantly impacting female welfare (Jørgensen, 2000; Fabà et al., 2018). Locomotion problems and lameness are more frequently cited reasons for culling in high-parity sows (Iida et al., 2020), often exacerbated by their heavy body weight. However, these culling reasons are also frequent for gilts and primiparous in commercial farms (Wang et al., 2019) and for gilts served then culled in gilt development units (Ulguim et al., 2014). Lame females tend to have low weaned litter size and prolonged WEI, which further elevate their risk of subsequent culling (Iida et al., 2020). The incidence of these conditions may increase in gilts raised in group housing systems due to disputes and aggressions, but it may be minimized by reducing the mixing groups of gilts (Verdon et al., 2015), a practice more likely to occur in herds with optimized health status (Supakorn et al., 2017; Iida et al., 2019). Since overweight gilts are prone to delayed puberty, older at the time of the first mating (Schenkel et al., 2010; Koketsu and Iida, 2020) and may continue to be overweight subsequently, strategies to induce puberty and control weight gain in gilts are crucial to minimize NPD and premature culling.

Recent observational data have reported increased female mortality rates (Monteiro et al., 2022; Paiva et al., 2023), which impacts reproductive longevity. Although the risk of mortality increases with parity, it can be high for gilts and primiparous (Sasaki and Koketsu, 2008). As mortality is multifactorial and can only be assessed post-mortem, preventing risk factors for female deaths may be difficult. Sudden deaths constitute nearly one-third of all female deaths in large production systems (Paiva et al., 2023), frequently attributed to acute health conditions that can occur across all parities (Monteiro et al., 2022). Nevertheless, the risk of sudden death due to heart failure is increased for females with a body condition score greater than 3.5 (Monteiro et al., 2022), which may be common in over-conditioned gilts.

An increased occurrence of pelvic organ prolapses (uterine, vaginal, and rectal) has been reported concurrently with the rise in female deaths (Supakorn et al., 2017; Kiefer et al., 2021a), primarily attributed to events related to gestation and parturition. Displacements in uterosacral and cardinal ligaments and disturbances in pelvic floor dynamics were identified as risk factors according to animal models used to investigate such prolapses in women (Easley et al., 2017). The incidence of prolapses, particularly uterine prolapses, is more common in sows at higher parities (Iida et al., 2019; Bhatia et al., 2023), attributed to decreased muscle and uterine tone. However, the likelihood of vaginal and rectal prolapses in both nulliparous and primiparous may be similar to or even greater than that in multiparous sows (Supakorn et al., 2017; Iida et al., 2019). This may be because young females have less mature muscle and supporting tissue around the anus area (Iida et al., 2019).

Prolapses are usually recorded within the peripartum and post-farrowing periods (Iida and Koketsu, 2014), as primiparous surviving prolapses occurring during lactation are often culled after weaning and are not re-mated (Supakorn et al., 2017). Although there is a common belief that farrowing induction might increase the risk of prolapse-related deaths, a comprehensive study did not confirm this association (Iida et al., 2019). Nevertheless, the incidence of prolapses is linked to excessive and incorrect manual obstetric interventions (Monteiro et al., 2022). Litter size may be a risk factor for rectal prolapses, since large litters are associated with prolonged gestation length, increased abdominal pressure during parturition, and a high stillbirth rate (Vanderhaeghe et al., 2013; Iida et al., 2019). Farrowings with at least two stillborns are more likely to result in rectal prolapses compared to those with no stillbirths. However, no associations of stillbirths with uterine and vaginal prolapses were found (Iida et al., 2019). In contrast, small litters, which may also be frequent in primiparous, may also be related to increased risk of prolapses, as small litters commonly include large heavy piglets that may cause obstruction and trauma in the birth canal, leading to difficult deliveries (Iida et al., 2019).

Females at risk of pelvic organ prolapses exhibit specific vaginal microbiota, distinct from those of females that did not experience prolapses (Kiefer et al., 2021a). Moreover, during late gestation, females at high risk of prolapses show different serum concentration of reproductive steroid hormones and certain metabolic biomarkers compared to those at reduced risk (Kiefer et al., 2021b). As estradiol and progesterone regulate inflammatory and immune responses induced by these biomarkers (García-Gómez et al., 2020), this knowledge may aid in developing therapies to prevent prolapses and treat genitourinary pathologies (Kiefer et al., 2021b). A GWAS identified several candidate genes associated with susceptibility to pelvic organ prolapses (Bhatia et al., 2023), reporting a moderate heritability of such prolapses across parities, which declines as parity increased. As the highest by-parity heritability was observed for parity-two sows, gilts and primiparous females are promising targets for genetic selection aimed at mitigating the impact of prolapses.

## Lifetime productivity: how much is enough?

Annual reproductive efficiency, expressed by the number of PW/F/Y, is determined by fertility and prolifacy (Dial et al., 1992). Furthermore, keeping productive females longer in the breeding herd is of interest, to optimize lifetime productivity. Although it has been traditionally expressed by the number of parities at removal (Patterson and Foxcroft, 2019; Iida et al., 2020), female lifetime productivity also depends on reproductive longevity, in addition to fertility and prolifacy.

Reproductive longevity is not necessarily represented by the number of female life days, which considers the period from birth to removal, including periods when gilts were not yet in the breeding herd. Instead, reproductive longevity can be expressed by the number of female herd days (Lucia et al., 1999b; Saito et al., 2011; Koketsu et al., 2020). Prior to the first service, all herd days are NPD since gilts are not yet gestating. After service, the herd days become potentially productive. However, if certain subsequent conditions lead to premature removal, all preceding herd days revert to NPD, as those females would contribute with no piglet output (Lucia et al., 2000b; Engblom et al., 2007). For gilts that do not make it to the first farrowing, the NPD may correspond to as much as four months (Lucia et al., 2000a). In segregated development units supplying pregnant replacement gilts, culling can occur after nearly 72 herd days for those non-served and with 107 herd days for those served then culled (Ulguim et al., 2014).

Over the years, annual culling rates have remained high and the average parity at removal ranged between 4.5 and 5.0 (Iida and Koketsu, 2015; Iida et al., 2020; Koketsu et al., 2020). Nevertheless, as time has passed, females have changed. Currently, they reach puberty earlier, farrow larger litters, and are re-bred after a short WEI. With a 0.2-unit annual increase in the ovulation rate (Kemp et al., 2018), sows weaning nearly 40 piglets per year may soon become old news (Koketsu and Iida, 2020). As the number of PW/F/Y can be constantly monitored, risk factors impacting reproductive efficiency can be identified on a timely basis. However, assessing female lifetime productivity requires analyzing retrospective data. And it takes time!

Female removal is costly. For removed females, the usual maintenance expenses (such as feed, labor, medication, fixed facility costs, etc.) are compounded by additional opportunity costs during the NPD (Dial et al., 1992), which are proportional to their herd days (Lucia et al., 2000b). For removed gilts, the only revenue is their market value since they have not farrowed any piglets. Gilts that became primiparous generate greater revenues, as they have farrowed and weaned one litter, but remain at risk of culling after weaning, in the event of subsequent reproductive failures or health conditions that would increase their NPD (Iida and Koketsu, 2015). Therefore, female lifetime productivity should consider both reproductive longevity and cumulative prolifacy, expressed by the total number of pigs born/weaned per female until removal (Lucia et al., 1999b; Koketsu et al., 2017; Koketsu and Iida, 2020).

Within each parity, the retention rate reflects the frequency of females reaching the next parity over the total number of females from the same parity that were previously served (Tani et al., 2016). For instance, the retention of primiparous would be increased if selection for litter size were to occur only after two parities (Thingnes et al., 2015; Wülbers-Mindermann et al., 2015). Primiparous females begin accumulating their piglet output in the first parity like a savings account, which may receive new deposits at subsequent parities, at varying intervals depending on the NPD accumulated in the meantime. Thus, as females extend their reproductive longevity, their cumulative piglet output may gradually offset the investments made in their maintenance up to that period, making it more likely to break even the costs incurred during their herd days (Rodriguez-Zas et al., 2003). Paradoxically, their likelihood of culling may increase, as their market value declines and their piglet output is unlikely to significantly increase, making replacement by a younger gilt more profitable (Koketsu et al., 2020; Rodriguez-Zas et al., 2003). Therefore, determining how many parities (or piglets) are necessary for a replacement gilt to reach the break-even point is complex. Herds with similar culling rates may exhibit distinct culling patterns and risk factors for impaired performance may reflect events that occurred long ago. Additionally, female lifetime productivity is multifactorial and has low heritability (Serenius and Stalder, 2006; Wijesiriwardana et al., 2022) and the economic context of the evaluation period changes over time (Rodriguez-Zas et al., 2003).

Some studies suggest that a sow should produce at least three litters before removal from the herd (Sasaki and Koketsu 2008; Patterson and Foxcroft, 2019). For a female from nearly three decades back, this would translate to a herd life slightly shorter than two years, with nearly 41 born alive and 36 weaned pigs (Lucia et al., 2000b). In today’s highly productive herds, reaching three parities at removal would occur in a little over two years, during which the lifetime output would amount to more than 70 born alive and 61 weaned pigs (Koketsu et al., 2020). However, with continuous annual increase in ovulation rate by 0.2 units (Kemp et al., 2018), such output might either accumulate over a shorter lifetime or be greater even with similar reproductive longevity. Regardless, culling low-parity sows with fewer piglets produced is not good business, since the costs incurred during their herd days would not be paid off (Rodriguez-Zas et al., 2003). In contrast, other studies suggest that cost-effective reproductive longevity would require at least five parities at removal (Huirne et al., 1991; Kristensen and Søllested, 2004). That emphasizes that various risk factors for premature female removal may be interconnected, having distinct impacts depending upon herd-specific contexts. In herds with high reproductive efficiency, females with low lifetime productivity may accumulate fewer NPD than those with high lifetime productivity (Takanashi et al., 2011) since their poor performance at early parities might lead to their removal with shorter reproductive longevity. In such cases, the performance of a replacement gilt may be expected to be greater compared to that of a retained sow, which would likely continue to exhibit poor reproductive efficiency.

## Conclusions and future implications

Pig farms present increased reproductive efficiency thanks to improvement in ovulation rate and litter size but require a constant supply of replacement gilts due to persistently high female culling rates. Gilts reach puberty between 150-180 d of age and are first mated when they reach a liveweight of 135-150 kg, usually at their second or their third estrus. Gilts that are too old at the first mating are at risk of premature removal. Premature culling is primarily driven by reproductive failures and locomotor problems, while an increased mortality rate has also been recently reported. Sows with prolonged reproductive longevity are often characterized by farrowing large litters in their first two parities. Determining the cost-effective number of parities at removal should consider the cumulative number of pigs born and weaned during the herd life and the potential future productivity of a replacement gilt.
